# The Present Status of Immuno-Oncolytic Viruses in the Treatment of Pancreatic Cancer

**DOI:** 10.3390/v12111318

**Published:** 2020-11-17

**Authors:** Scott D. Haller, Michael L. Monaco, Karim Essani

**Affiliations:** Laboratory of Virology, Department of Biological Sciences, Western Michigan University, Kalamazoo, MI 49008, USA; scott.haller@crl.com (S.D.H.); michael.l.monaco@wmich.edu (M.L.M.)

**Keywords:** viruses, immuno-oncolytic viruses, pancreatic cancer

## Abstract

Pancreatic ductal adenocarcinoma (PDAC) is the fifth leading cause of cancer-related death in Western countries. The incidence of PDAC has increased over the last 40 years and is projected to be the second leading cause of cancer death by 2030. Despite aggressive treatment regimens, prognosis for patients diagnosed with PDAC is very poor; PDAC has the lowest 5-year survival rate for any form of cancer in the United States (US). PDAC is very rarely detected in early stages when surgical resection can be performed. Only 20% of cases are suitable for surgical resection; this remains the only curative treatment when combined with adjuvant chemotherapy. Treatment regimens excluding surgical intervention such as chemotherapeutic treatments are associated with adverse effects and genetherapy strategies also struggle with lack of specificity and/or efficacy. The lack of effective treatments for this disease highlights the necessity for innovation in treatment options for patients diagnosed with early- to late-phase PDAC and immuno-oncolytic viruses (OVs) have been of particular interest since 2006 when the first oncolytic virus was approved as a therapy for nasopharyngeal cancers in China. Interest resurged in 2015 when T-Vec, an oncolytic herpes simplex virus, was approved in the United States for treatment of advanced melanoma. While many vectors have been explored, few show promise as treatment for pancreatic cancer, and fewer still have progressed to clinical trial evaluation. This review outlines recent strategies in the development of OVs targeting treatment of PDAC, current state of preclinical and clinical investigation and application.

## 1. Introduction

Pancreatic cancer has one of the poorest prognoses of all common cancers, with under a 10% five-year survival rate [1]. Pancreatic ductal adenocarcinoma (PDAC) is the most common form of pancreatic cancer, accounting for about 90% of all cases of pancreatic cancer [2,3,4]. By the year 2030, PDAC is expected to become the second leading cause of cancer-related deaths in the United States [5,6]. The primary reason for the low survival rate of PDAC is a lack of direct or indirect diagnostic biomarkers for the disease which leads to late-stage diagnosis [2]. Most patients have either locally advanced or metastatic disease by the time of detection and diagnosis, which prevents curative resection [3]. Since complete resection is currently the only potential cure for PDAC, early detection is critical in the pursuit of increasing the median survival length of PDAC patients. One method of achieving early diagnosis is to screen patients demonstrating risk factors associated with PDAC.

Genetic risk factors, or non-modifiable risk factors, are associated with causation in 5–10% of new cases of PDAC [7,8]. Lifestyle-related risk factors, or modifiable risk factors, which include smoking, obesity, alcohol abuse and diabetes have also been linked to PDAC [9]. In the United States, 16.9% of PDAC cases can be attributed to body weight/obesity, while 10.2% can be attributed to smoking [10]. The aforementioned lifestyle-related risk factors are highly correlated with the occurrence of PDAC. Life-style associated risk factors are inherently modifiable; many can be mitigated or eliminated through patients electively executing life-style modifications, such as improved diet, frequent exercise and elimination of smoking. Medical intervention can also be implemented by primary care physicians. For example, statins show promise in their ability to potentially reduce the risk of developing PDAC associated with obesity or high cholesterol, especially in males [11]. Commitment to risk-mitigating lifestyle habits may reduce incidence of PDAC, especially in populations inherently vulnerable such as the elderly or those who have genetic risk factors [7,8,9]. Screening of vulnerable populations for precancerous lesions may also assist in the prevention of PDAC via potentially improving early detection and diagnosis.

Following patient diagnosis, it is important to obtain proper staging of the disease to determine the most effective treatment regimen. Preoperative evaluations for the potential surgical resection are primarily facilitated via diagnostic imaging using computed tomography (CT) and/or magnetic resonance imaging (MRI); CT being the most common due to greater accessibility in clinical centers. However, due to spatial resolution and tissue density sensitivity limitations in CT (specifically related to difficulty in adequately resolving low density tissue borders such as those of the lymph node or blood vessels in close approximation to high density tissues), up to 20% of patients are staged incorrectly upon primary diagnosis [12]. 

The most commonly practiced contemporaneous treatment is, as previously noted, surgical resection with adjuvant chemotherapy. Currently, adjuvant therapy is recommended for all patients with R0 or R1 resected PDAC [13] which, since adoption as common practice in the 1990s, has demonstrated marked improvement in patient survival in combination therapy with gemcitabine and capecitabine [14]. It is possible, as well, that patients with borderline resectable PDAC upon diagnosis may become resectable if treated with neoadjuvant chemotherapy with the folinic acid, 5-fluorouracil, irinotecan and oxaliplatin (mFOLFIRINOX) regime [12]. Patients with non-resectable PDAC will most commonly undergo palliative therapy [15].

While these advancements in PDAC treatment regimens have improved patient survival rates, reoccurrence is found in 75% of patients within the first two years after resection [14]. Continued efforts must remain focused on continued improvement in patient outcome and potential inclusion of additional therapeutics or procedures to attain such. Future strategies may include modified combination therapy approaches to include therapeutic agents such as immuno-oncolytic viruses (OVs) which have demonstrated potential against differing forms of cancer and are currently being evaluated as treatment options against pancreatic cancer.

## 2. Current PDAC Treatment Regimens

This section will review contemporaneous clinical treatment regimens being employed to target PDAC including surgical intervention, chemotherapeutic regimens and combination therapies.

### 2.1. Resectable PDAC

In patients with early stages of disease (stage I or II), successful resection may be possible [16]. However, even with resection in patients presenting with R0 tumor state, up to 70% of patients experience recurrent disease [17]. Due to the high risk of recurrence, adjuvant chemotherapy is recommended for all resected patients as combination treatment regimen with resection [15]. In order to help prevent recurrence, R0 resection is necessary [12]. A true R0 resection means that there are no tumor cells within 1 mm of the resection margin [3]. This is also sometimes called “R0 wide” resection, while a resection with tumor cells present within 1 mm of the resection margin but not within the margin itself is sometimes called “R0 narrow” resection [3]. A resection that has tumor cells within the resection margin itself is called an R1 resection [3]. As would be predicted by increased probability of incomplete resection of all cancerous cells in cases in which margin between tumor and healthy tissue is narrow or non-existent, patients treated via R0 wide resection have better prognosis than R0 narrow or R1 patients [3]. After resection, chemotherapeutic agents are delivered locally or systemically to increase the median length of patient survival; contemporaneous treatment regimen entails 6-month adjuvant chemotherapy with modified folinic acid, 5-fluorouracil, irinotecan and oxaliplatin (mFOLFIRINOX) in patients determined suitable. In patients determined to be at greater risk due to stress of the mFOLFIRINOX protocol, 6-month treatment via gemcitabine and capecitabine are followed based on the European Study Group for Pancreatic Cancer (ESPAC)-4 study [13]. Standards of care differ between differing populations and/or geographical regions. For example, the Japanese standard of care is to treat PDAC patients with S-1, an oral 5-fluorouracil (5-FU) prodrug. S-1 is designed to improve the antitumor activity of 5-FU by inhibiting dihydropyrimidine dehydrogenase, the key enzyme of 5-FU catabolism [18]. However, standard treatment for Western patients’ is either mFOLFIRINOX and/or a combination of gemcitabine and capecitabine; treatment selection being overall patient-health status dependent [13].

### 2.2. Borderline Resectable PDAC

PDAC may be defined as borderline resectable under differing anatomical presentations primarily as a function of organs/tissues adjacent to or in contact with the primary tumor. Cases defined as borderline resectable include those in which the tumor may contact the superior mesenteric artery at an angle greater than 180 degrees or the tumor may contact the celiac trunk [12]. However, a commonality shared amongst the majority of borderline resectable cases is successful, complete resection. There is some controversy over what constitutes borderline resectability in PDAC and case inclusion in determination of success rates associated with treatment of this presentation [12].

### 2.3. Neoadjuvant Chemotherapy

Patients with borderline resectable PDAC may undergo neoadjuvant chemotherapy intended to retract the tumor border from surrounding tissues resulting in a tumor presentation consistent with those classified as R0 or R1, thus the patient being a candidate for resection [12]. Chemotherapeutic regimens such as mFOLFIRINOX and combination therapy with gemcitabine and capecitabine have both shown promise as neoadjuvant treatment of PDAC in borderline resectable patients to increase the number of patients considered suitable for undergoing R0/R1 resection of the tumor [12]. The mFOLFIRINOX treatment regimen is considered to be an aggressive approach and although it can increase the likelihood of resectability, toxicity of the mFOLFIRINOX regimen limits its applicability to those patients considered to be able to withstand the greater potential for adverse side-effects [17]. Neoadjunctive chemotherapy in patient populations unsuitable for mFOLFIRINOX are treated with gemcitabine and capecitabine most commonly [17]. Determination of the success of neoadjunctive treatment is confounded in many cases due to the spatial resolution limitations of soft tissue via commonly applied imaging techniques, primarily CT. The intention is to provide diagnostic assessment of progression toward an R0 or R1 presentation. Often, upon direct visual inspection of the tumor and surrounding tissues during surgical resection, cases are discovered of incomplete regression of the tumor border [3]. 

For the reasons discussed above and the time-sensitive nature of the progression of PDAC, it has been recommended by some that patients who have undergone neoadjuvant chemotherapy should undergo exploratory surgery with intended R0–R1 resection with or without confirmatory diagnostic assessment via preoperative imaging, as long as they show no clear signs of disease progression [12]. It is important to note that while neoadjuvant chemotherapeutic treatment of borderline resectable PDAC has increased rates of R0 resection and growing data supports the beneficial effects of this approach on patient survival rates, most of this data consists of retrospective analysis. It remains necessary to rigorously assess the application of neoadjunctive chemotherapy through controlled, randomized clinical trials to determine best practice [12].

### 2.4. Chemotherapy for Non-Resectable PDAC

The previously discussed PDAC cases in which multi-paradigm treatment approaches of surgical resection and neo- or adjunctive chemotherapy are applied represent only ~10–20% of all PADC cases. The majority of PDAC cases, 80–90%, are comprised of patients presenting with locally advanced, non-resectable tumors and systemically disseminated metastases [19,20]. In locally advanced and metastatic PDAC, systemic chemotherapy is employed as the standard of care to extend the life of the patient [13]. Consistent with neo- and adjunctive chemotherapeutic regimens applied in resectable PDAC, the standard chemotherapeutic regimens for advanced PDAC also consist of nucleoside analogues, including gemcitabine and capecitabine, or the pyrimidine analogue 5-FU as monotherapies or in combination the mFOLFIRINOX regimen. Combination therapy has been reported to nearly double median survival in the metastasized stage as compared to gemcitabine alone, and the combination of gemcitabine and a nanoparticle albumin-bound paclitaxel (nab-paclitaxel) has also been shown to significantly improve overall survival [21,22]. However, as previously noted, these protocols are associated with relevantly higher toxicity, thus often preventing their application in high-risk patients, but overall quality of life was reported to increase [17]. Systemic chemotherapy may also help to decrease the size and border architecture of the primary tumor, increasing probability for patient treatment via R0–R1 resection which would increase likelihood of patient survival [12]. 

The application of radio-chemotherapy is relatively infrequent in treatment of PDAC given the high incidence of primary diagnosis being late-stage disease classification. This limits the value of local treatment procedures. Furthermore, PDAC demonstrates resistance to radio-chemotherapy, which is currently addressed by combining PDAC radiotherapy with radiosensitizing agents, including gemcitabine, capecitabine, or 5-FU, respectively. The National Comprehensive Cancer Network (NCCN) has recently released guidelines recommending radio-chemotherapeutic treatment of PDAC patients with borderline-resectable tumors resulting in moderate, yet increasing, clinical application [23].

### 2.5. Palliative Surgery to Improve Quality of Life (QOL)

Patients with PDAC often suffer from conditions which are commonly addressed through palliative surgery to improve quality of life (QOL). The three primary symptoms leading to palliative surgical application in PDAC patients are duodenal or gastric outlet obstruction (GOO), obstructive jaundice (OJ) and pain [15]. GOO is treated surgically, often with an open gastrojejunostomy (GJ) [15]. Laproscopic GJ has recently been developed as an option for the treatment of GOO, and it shows promise with fewer negative side effects, but further studies are needed to confirm its feasibility [15]. Endoscopic placement of a biliary stent is the current treatment for palliation of OJ resulting from PDAC [15]. Patients with unresectable or incurable disease found during exploration (11–50%) are generally considered to be best treated with surgical palliation with demonstrated improved QOL [24].

## 3. Introduction to Oncolytic Viruses

This section will present the genesis of development and application of immune-oncolytic viruses in the treatment of various forms of cancer and focus on the current application of OVs in the treatment of pancreatic cancer.

### 3.1. Definiton of Oncolytic Virus

**Figure 1:**
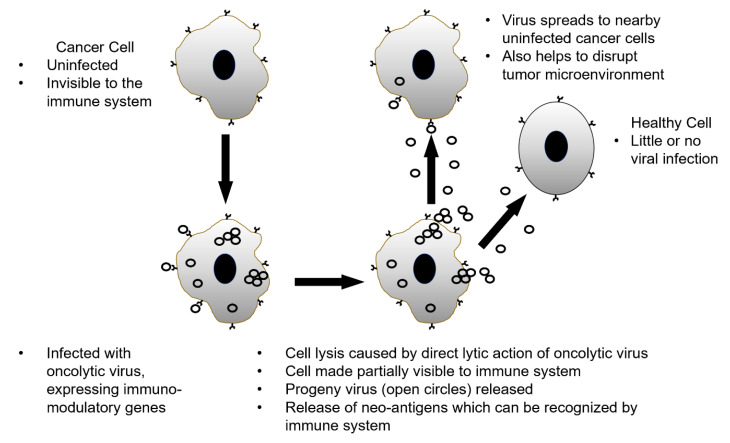
Simplified pathways oncolytic viruses employ to potentially regress tumors.

The term “oncolytic virus” (OV) originated following the discovery of potential use and subsequent application of differing naturally occurring or genetically modified viruses as therapeutic agents in the treatment of various forms of cancer. Typically, these are non-pathogenic viral strains that demonstrate differing modes of selectivity for replication in cancer cells over noncancerous cells [25,26,27,28]. As standard practice for development of novel therapeutics, OVs have been assessed and have demonstrated efficacy in regression of differing forms of cancer in preclinical models [29]. The mechanism of action (MOA) of OVs differ widely: such as direct malignant cell lysis, expression of cytotoxic or immunomodulatory genes and inherent susceptibility of differing forms of cancer to viral replication [25,26,30]. The approach of genetically modifying wild-type virus to express immunomodulatory genes resulting in stimulation or suppression of the patient’s immune system results in an immunogenically “hot” environment around the tumor, which promotes regression of the malignant cell population [31]. OV expression of immunomodulatory genes and resultant malignant cell regression may occur through differing mechanisms dependent on the gene(s) expressed, such as direct cell lysis, disruption of tumor microenvironment vasculature or other, ultimately leading to destruction of cancer cells [32]. A representation of how OVs can potentially eliminate tumors is shown in Figure 1. As previously noted, specific OVs can be either genetically modified to selectively target and replicate in cancer cells, or OV can be used to target known disruptions in normal cell anti-viral activity in malignant cells. For example, in various forms of cancer the interferon signaling pathway is disrupted which results in decreased protein kinase R (PKR) activity; PKR is an intracellular protein kinase which recognizes double-stranded RNA and other viral elements leading to cell death and clearance of the virus. In some cancerous cells, the PKR signaling cascade is disrupted, this allows for viral replication to proceed uninterrupted and lead to effective regression of cancer cell population [33].

### 3.2. History

The first recorded correlation of a naturally occurring viral infection and potential anticancer activity was discovered in 1904 when Dr. George Dock published a report about a patient with leukemia who experienced a decreased leukocyte count after a naturally occurring influenza virus infection [34]. In 1949, the Russian Far East Virus was observed to inhibit the growth of tumors transplanted into mice [35]. In 1960, opportunistic infection of the rat protoparvovirus, H-1PV, was shown in transplantable human tumors, subsequently H-1PV was directly assessed in preclinical models and demonstrated suppressive properties of tumors cell proliferation [32]. These and other such discoveries of the potential anticancer properties of virus and advancements in recombinant DNA technology led to the development of genetically modified forms of virus to leverage the natural properties of viral replication in host cells as a means to induce cancer cell death and/or other means of regressing tumor progression via deletion or insertion of specific genes of interest to recruit the patient’s antitumor immunity. In 1991, a genetically modified form of herpes simplex virus (HSV) was developed with depleted thymidine kinase or infected cell protein 34.5 which demonstrated preferential replication of the virus in human glioma xenografts [36]. In 2006, H101, a genetically altered adenovirus serotype-5, was approved in China for the treatment of nasopharyngeal cancers [37]. Following approval of H101 in China, in 2015, T-Vec, a modified HSV, was approved in the United States for the treatment of advanced melanoma [38]. Since the approval of T-Vec by the US Food and Drug Administration (FDA) and European Medicines Agency (EMA), the use of OV as an established immunotherapy has become more widely adopted with significantly increased clinical trial activity assessing OVs and their application as a monotherapy or combination therapy against differing forms of cancer. A selection of ongoing clinical trials is summarized in Table 1.

### 3.3. Oncolytic Viruses as Pancreatice Cancer Therapeutic 

As previously discussed, forms of pancreatic cancer, specifically PDAC, maintain several distinct characteristics that present challenges for most all therapeutic approaches. One such characteristics of PDAC is the composition of the tumor microenvironment presenting as dense fibrotic stroma and stellate cells which prevent or inhibit access of intended therapeutic agents to proliferating cells. Furthermore, the PDAC microenvironment is known to express immunosuppressive factors such as transforming growth factor-beta (TGF-β), interleukin-10 (IL-10) and others [43]. PDAC tumors also do not express neoantigens, thus immune system response to the tumor is limited [44]. As a result of these characteristics, treatment strategies such as those previously described present unique challenges for therapeutic efficacy of OVs. The density and impenetrability of the PDAC tumor microenvironment limit access to and prevent robust exposure of OVs to the proliferating PDAC cells. To overcome the specific challenges associated with the density of the PDAC microenvironment, the use of Vitamin D in conjunction with therapeutic agents or the use of hyaluronidase is currently being investigated for the potential to increase OV exposure to the tumor microenvironment and facilitate enhanced therapeutic efficacy [31,45]. One can also predict the challenges posed to other common strategies of immune system modulation through viral-induced transfection being limited due to PDAC microenvironment immunosuppression and lack of neoantigen expression.

Below, we present several approaches and the current status of PDAC treatment via differing families of OVs.

### 3.4. Adenovirus

A significant number of ongoing research programs assessing the potential for OV platforms as a treatment of PDAC are comprised of those using adenoviruses (AV). The focus on use of AVs is due to several factors including high endogenous presence in the human population, DNA as viral nucleic acid, high transfection efficiency, low probability of insertional mutagenesis and suitability for genetic-modification dependent cancer type specificity. Of the 57 serotypes of AVs, AV5 was the first to be investigated [46] and as previously mentioned, the H101 platform was approved in 2006 for treatment of nasopharyngeal cancer in China [37]. As an AV-5 serotype, H101, as shared with other AV-5 AVs, demonstrates natural tropism to respiratory tissue, as such, have limited applicability in the potential treatment of PDAC or GI cancers. Contemporaneous OV development programs focus to leverage the natural tropism of OVs for the intended type of malignancy, thus AV-12, AV-40, AV-41 and AV-52 serotypes stand out as natural candidates for investigation into their effects on GI malignancies due to known tropism for GI tissues [46]. 

ONYX-015, an AV-5 OV therapy, which replicates selectively in cells demonstrating p-53 mutations and dysfunction, failed to cause an objective response when used as a monotherapy [36]. Under Phase II clinical trial assessment ONYX-15, when used in combination with gemcitabine, a limited response was observed in early clinical trials, but issues associated with low viral replication and patient development of high titer neutralizing antibodies resulted in cessation of the trial [31,36]. 

Leveraging the results of and understating the challenges encountered through the ONYX-015 trials, current attempts to increase the effectiveness of potential adenovirus vectors for PDAC treatment focus on genetic engineering of the virus to develop variants specific to the disease characteristics with the hopes of improving efficacy. Examples of such are the adenovirus AxE1AdB-UPRT which expresses uracil phosphoribosyltransferase (UPRT), which helps overcome 5-FU resistance [46]. The AV-5 vector AV-5-yCD/mutTkSR39rep-ADP carries AV cytosine deaminase and HSV thymidine kinase and it was shown to improve the efficacy of radiotherapy in preclinical models [47]. VCN01, an AV modified to express hyaluronidase among other modifications, was tested in a preclinical model to adjust the tumor microenvironment to make PDAC more susceptible to OV therapy [46,48]. The VCN-01 and LOAd703 oncolytic adenoviruses have moved to Phase I trials as monotherapies or in combination with paclitaxel/gemcitabine. As can be seen in Table 2, there are many active clinical trials focusing on AV vectors in the treatment of PDAC. Researchers continue to explore the use of the adenovirus platform as a potential treatment of PDAC both for intratumoral and intravenous administration.

### 3.5. Herpes Simplex Virus

With the success of T-Vec and its approval for treatment of nasopharyngeal cancer, HSVs seem like a promising OV platform for further development and refinement. T-Vec is currently being assessed for potential efficacy in the treatment of melanoma, Merkel cell carcinoma and other forms of solid-state tumors in a Phase I clinical trial as monotherapy or in combination with radiotherapy (NCT02819843) [38]. T-Vec has also been assessed directly as a monotherapy against PDAC in a phase I trial, results demonstrated no objective response was found in 17 patients enrolled [40]. Myb34.5, a genetically altered, replication-conditional recombinant HSV which leverages the known overexpression of the cellular B-*myb* promoter in PDAC cells has been assessed in preclinical models. The Myb34.5 HSV variant, intratumorally injected and as a monotherapy it inhibited the growth of PDAC tumors and induced apoptosis. This result was also found to the enhanced in a dose-dependent manner in combination therapy with gemcitabine [49].

Unlike the human-engineered genomic variations of the T-Vec and Myb34.5 oncolytic HSVs, HF10 is a natural, spontaneously mutated HSV variant; the well-characterized mutations of HF10 and assumed resultant, potentially favorable, oncolytic activity have been assessed against many differing forms of cancer with positive results in preclinical models with and without combination therapy with chemotherapeutic agents [30]. A phase I clinical trial was conducted in eight, male Japanese PDAC patients who were HSV seropositive to assess efficacy and safety of HF10. Results showed no adverse effects across all patients with 3 of 8 (37%) of patients demonstrating reduced levels of the PDAC tumor marker CA19-9. Furthermore, HF10 envelop proteins were detected in autopsy specimens, as were macrophages, CD4+ and CD8+ cells and markers of natural killer (NK) cell activation. These results suggest that higher doses of HF10 can be used in clinical trials moving forward and HF10 may enhance antitumor immune system activity [30]. A current study combines HF10 with chemotherapeutic agents and is summarized in Table 2 (NCT03252808).

### 3.6. Protoparvovirus

H-1PV is a rodent protoparvovirus originally isolated from transplantable human tumors and found to be an opportunistic virus with natural tropism for human cancer cells. H-1PV has also been shown to mitigate spontaneous tumor formation in preclinical models [50,51]. While H-1PV has been observed in human tumors, it does not naturally occur in humans, thus allowing for primary treatments to be less negatively impacted by the patient’s immune system clearance of the virus. This increases the duration of the therapeutic window for application as an OV as compared to other OVs endogenous in the human population [32]. As with other OVs, H-1PV, when used as combination therapy with gemcitabine, increased median length of survival in preclinical models [52]. ParvOryx02 was a phase I/IIa clinical study using H-1PV in PDAC patients to test for tolerability and safety (NCT02653313). While the study has been completed, results have yet to be formally published. The Principal Investigator (PI) of the ParvOnyx02 trial- Guy Ungerechts; recently reported positive results of the trial have been reported at the Oncolytic Virus Immunotherapy Summit and International Oncolytic Virus Conference in 2019 indicating H-1PV was well tolerated in all patients and 2 of 7 patients experienced prolonged survival times which were associated with favorable immunological signatures.

### 3.7. Reovirus

Reoviruses are double stranded RNA viruses which replicate only in cells with an activated retrovirus-associated DNA sequences (RAS) pathway. A hallmark isoform mutation of PDAC is the mutation of Kirsten-RAS (KRAS), as such reovirus has a natural tropism for PDAC cells [53]. Due to reovirus tropism for PDAC, this family of virus has been assessed for potential as PDAC therapeutic, preclinical assessments demonstrated induction of apoptosis in PDAC tumors due to endoplasmic reticulum stress; clinically, Pelareorep, an isolate of a strain of reovirus, failed to increase progression-free survival either as monotherapy or in combination with paclitaxel and carboplatin [36,41]. In a separate phase II clinical study, Pelareorep was assessed as combination therapy with gemcitabine, results showed efficient viral replication within tumor cells with the combination therapy being was well-tolerated. Unfortunately, though, there was no significant benefit to the combination versus gemcitabine alone [54]. The potential of reovirus, specifically Pelareorep, continues ongoing clinical assessment as summarized in Table 2. Results recently published in 2020 from a phase Ib single-arm study comprised of patients with PDAC who progressed after first-line treatment demonstrated Pelareorep and pembrolizumab added to chemotherapy did not add significant toxicity and showed encouraging efficacy [55].

### 3.8. Vaccinia Virus

Vaccinia virus (VV) is a double-stranded DNA virus which is commonly used as a vaccine, most notedly as smallpox vaccine, and others due to suitability for genetic modification [56]. Amenability of VV for genetic modification and other favorable characteristics enabling potential use as an OV have led to assessment of VV variants against various forms of cancer. For example, JX-594 is a genetically engineered VV assessed for efficacy against hepatocellular carcinoma demonstrating well tolerated treatment and appreciable transgene expression and systemic dissemination in human patients [57]. As with previously described OVs, observed oncolytic activity against one form of cancer inevitably leads researchers to investigate the potential use of the specific OV against various forms of cancer. As such, the replication-competent VV variants GLV-1h68 and GLV-1h151 were assessed against pancreatic cancer cells maintained in in vitro and in vivo environments. Results demonstrated GLV-1h68 was effective as monotherapy and enhanced in combination treatment with gemcitabine and cisplatin [58]. GLV-1h151 also demonstrated efficacy as monotherapy, efficacy being enhanced when applied in combination with radiotherapy [59]. Multiple variants of VV have also been assessed against pancreatic cancer in preclinical studies leveraging the virus as an immunomodulatory agent and vaccine, results vary, but all provide greater insight as to the potential for VV as a treatment platform against human PDAC [60]. Clinical assessment of VV efficacy against PDAC has been limited. A Phase I study of VVDD in eleven patients presenting with various types of cancers, including PDAC, showed no dose-limiting adverse events related to treatment [61]. Currently, there are three ongoing clinical trials, represented in Table 2, involving VV as a vaccine in combination treatment with sargramostim in nonresectable PDAC patients (NCT00669734), as vaccine in combination therapy with pembrolizumab in PDAC patients failing previous treatments (NTC02432963) and as neoadjunctive treatment with variant GL-ONC1 prior to surgery (NTC02714374). All three trials are currently active, not recruiting as of last available updated through the U.S. National Library of Medicine.

## 4. Conclusions

The clinical application of naturally occurring or genetically modified viruses as potential cancer therapeutics has proven to be a successful strategy considering the approval of H101 and T-Vec for treatment of human patients with nasopharyngeal cancer and melanoma, respectively.

The use of OV as an established immunotherapy has become more widely adopted with significantly increased clinical trial activity assessing OVs and their application as a monotherapy or combination therapy against differing forms of cancer. The potential to apply a patient-specific panel of differing OVs in combination with traditional chemotherapy or other forms of treatment presents a very interesting and encouraging path forward for continued and increased scope of use of OVs in the treatment of differing forms of cancer. PDAC continues to pose specific challenges for the use of OVs as primary treatment platform, as well as traditional treatment paradigms as discussed throughout this review. Encouraging preclinical and clinical outcomes continue to support viral platforms such as vaccinia, reovirus, herpes simplex-1 and adenovirus as potential OVs in the treatment of PDAC. Continued investigation into the use of these and other OV platforms against PDAC is warranted given the promising results. To attain greater oncolytic efficacy, future investigations must be fundamentally driven by understanding the challenges specific to PDAC and designing OV platforms and combination treatment regimens specific to the disease characteristics of PDAC and potentially specific to individual patients.

## Figures and Tables

**Table 1:** A sample of completed clinical trials assessing immuno-oncolytic virus (OV) safety and efficacy.

Virus	Phase	Title	Interventions Used (OVs Italicized)	Enrollment Status	ClinicalTrials.gov Identifier
Adenovirus	I	*AdV-tk* + Valacyclovir Therapy in Combination with Surgery and Chemoradiation for Pancreas Cancer	*AdV-tk* Valacyclovir	Completed [39]	NCT00638612
Adenovirus	I	Phase I Study Combining Replication-Competent Adenovirus-Mediated Suicide Gene Therapy with Chemoradiotherapy for the Treatment of Non-Metastatic Pancreatic Adenocarcinoma	*Ad5-yCD/mutTKSR39rep-ADP*	Terminated (Poor enrollment)	NCT00415454
Adenovirus	I	A Phase I, Multicenter, Open-label, Dose Escalation Study of Intratumoral Injections of *VCN-01* Oncolytic Adenovirus with Intravenous Gemcitabine and Abraxane^®^ in Advanced Pancreatic Cancer	*VCN-01* Gemcitabine Abraxane^®^	Completed	NCT02045589
Herpes Simplex-1 (HSV-1)	I	A Phase I Study of Recombinant hGM-CSF Herpes Simplex Virus	*Recombinant HSV-1 Injection*	Completed	NCT01935453
Herpes Simplex-1 (HSV-1)	I	A Phase I Study of Repeated Intratumoral Administration of *TBI-1401(HF10)*, a Replication Competent HSV-1 Oncolytic Virus, in Patients with Solid Tumors with Superficial Lesions	*TBI-1401(HF10)*	Completed	NCT02428036
Herpes Simplex-1 (HSV-1)	I	Targeted Delivery of OncoVEX^GM-CSF by Endoscopic Ultrasound (EUS)-Guided Fine Needle Injection (FNI) in Patients with Irresectable Pancreatic Cancer: A Pilot Multinational Experiment on Safety and Proof of Concept	*Talimogene Laherparepvec*	Completed [40]	NCT00402025
Parvovirus	I/II	A Non-controlled, Single Arm, Open Label, Phase II Study of Intravenous and Intratumoral Administration of *ParvOryx* in Patients with Metastatic, Inoperable Pancreatic Cancer	*Parvovirus H-1*	Completed	NCT02653313
Reovirus	II	A 2-arm Randomized Phase II Study of Carboplatin, Paclitaxel Plus *Reovirus Serotype-3 Dearing Strain (Reolysin)* vs. Carboplatin and Paclitaxel in the First Line Treatment of Patients with Recurrent or Metastatic Pancreatic Cancer	Carboplatin Paclitaxel *Wild-type Reovirus*	Completed [41]	NCT01280058
Reovirus	II	A Phase 2 Study of *REOLYSIN* in Combination with Gemcitabine for Patients with Advanced Pancreatic Adenocarcinoma	*REOLYSIN* Gemcitabine	Completed	NCT00998322
Reovirus	I	A Phase 1b Study of Pembrolizumab (KEYTRUDA^®^) in Combination With *REOLYSIN*^®^ (Pelareorep) and Chemotherapy in Patients with Advanced Pancreatic Adenocarcinoma	*REOLYSIN* Gemcitabine Irinotecan Leucovorin 5-fluorouracil Pembrolizumab	Completed	NCT02620423
Vaccinia Virus	I	A Phase I Study of an MVA Vaccine Targeting P53 in Cancer	*Modified Vaccinia Virus* *ankara vaccine expressing p53*	Completed [42]	NCT01191684

**Table 2:** A sampling of current clinical trials involving pancreatic cancer and oncolytic viruses.

Virus	Phase	Title	Interventions Used (OVs italicized)	Enrollment Status	ClinicalTrials.gov Identifier
Adenovirus	I/IIa	Phase I/IIa Trial Evaluating Safety of *LOAd703,* an Armed Oncolytic Adenovirus for Pancreatic Cancer	*LOAd703* Gemcitabine nab-paclitaxel	Recruiting	NCT02705196
Adenovirus	I/II	NANT Pancreatic Cancer Vaccine: Molecularly Informed Integrated Immunotherapy Combining Innate High-affinity Natural Killer (haNK) Cell Therapy with Adenoviral and Yeast-based Vaccines to Induce T-cell Responses in Subjects with Pancreatic Cancer Who Have Progressed on or After Standard-of-care Therapy	Aldoxorubicin HCl ALT-803 ETBX-011 GI-4000 haNK avelumab bevacizumab Capecitabine Cyclophosphamide Fluorouracil Leucovorin nab-Paclitaxel lovaza Oxaliplatin Stereotactic Body Radiation Therapy	Active, not recruiting	NCT03387098
Adenovirus	I/II	Phase I/II Trial Investigating an Immunostimulatory Oncolytic Adenovirus for Cancer	*LOAd703* Standard of care chemotherapy	Recruiting	NCT03225989
Adenovirus	I	VISTA (Virus Specific T Cells and Adenovirus): A First in Human Phase I Trial of Binary Oncolytic Adenovirus in Combination with HER2-Specific CAR VST Cells in Patients With Advanced HER2 Positive Solid Tumors	*CAdVEC*	Not yet recruiting	NCT03740256
Adenovirus	I	A Phase I, Multicenter, Open-label, Dose Escalation Study of Intravenous Administration of *VCN-01* Oncolytic Adenovirus with or Without Gemcitabine and Abraxane^®^ in Patients with Advanced Solid Tumors	*VCN-01* Gemcitabine Abraxane^®^	Active, not recruiting	NCT02045602
Herpes Simplex-1 (HSV-1)	I	Phase I Study of Combination With *TBI-1401(HF1*0), a Replication-competent HSV-1 Oncolytic Virus, and Chemotherapy in Japanese Patients with Stage III or IV Unresectable Pancreatic Cancer.	*TBI-1401(HF10)* Gemcitabine Nab-paclitaxel TS-1	Active, not recruiting	NCT03252808
Reovirus	II	Pembrolizumab and *Pelareorep* in Treating Patients with Advanced Pancreatic Cancer	Pembrolizumab *Wild-type Reovirus*	Recruiting	NCT03723915
Vaccinia Virus & Fowlpox Virus	I	Immunotherapy for Unresectable Pancreas Cancer: A Phase 1 Study of Intratumoral Recombinant Fowlpox *PANVAC (PANVAC-F)* Plus Subcutaneous Recombinant Vaccinia *PANVAC (PANVAC-V)*, PANVAC-F and Recombinant Granulocyte-Macrophage Colony Stimulating Factor (rH-GM-CSF)	*Falimarev* *Inalimarev* Sargramostim	Active, not recruiting	NCT00669734
Vaccinia Virus	I	A Phase I Study of a *p53MVA* Vaccine in Combination with Pembrolizumab	*Modified Vaccinia Virus Ankara Vaccine Expressing p53* Pembrolizumab	Active, not recruiting	NCT02432963
Vaccinia Virus	I	An Open Label, Non-randomized Phase 1b Study to Investigate the Safety and Effect of the Oncolytic Virus *GL-ONC1* Administered Intravenously Prior to Surgery to Patients with Solid Organ Cancers Undergoing Surgery for Curative-Intent or Palliative Resection	*GL-ONC1*	Active, not recruiting	NCT02714374
